# Association of Exposure to Diagnostic Low-Dose Ionizing Radiation With Risk of Cancer Among Youths in South Korea

**DOI:** 10.1001/jamanetworkopen.2019.10584

**Published:** 2019-09-04

**Authors:** Jae-Young Hong, Kyungdo Han, Jin-Hyung Jung, Jung Sun Kim

**Affiliations:** 1 Division of Spinal Surgery, Department of Orthopedics, College of Medicine, Korea University, Seoul, South Korea; 2 Department of Biostatistics, College of Medicine, Catholic University, Seoul, South Korea; 3 Division of Hematology/Oncology, Department of Internal Medicine, College of Medicine, Korea University, Seoul, South Korea

## Abstract

**Importance:**

Diagnostic low-dose ionizing radiation has great medical benefits; however, its increasing use has raised concerns about possible cancer risks.

**Objective:**

To examine the risk of cancer after diagnostic low-dose radiation exposure.

**Design, Setting, and Participants:**

This population-based cohort study included youths aged 0 to 19 years at baseline from South Korean National Health Insurance System claim records from January 1, 2006, to December 31, 2015. Exposure to diagnostic low-dose ionizing radiation was classified as any that occurred on or after the entry date, when the participant was aged 0 to 19 years, on or before the exit date, and at least 2 years before any cancer diagnosis. Cancer diagnoses were based on *International Statistical Classification of Diseases and Related Health Problems, Tenth Revision* codes. Data were analyzed from March 2018 to September 2018.

**Main Outcomes and Measures:**

The primary analysis assessed the incidence rate ratios (IRRs) for exposed vs nonexposed individuals using the number of person-years as an offset.

**Results:**

The cohort included a total of 12 068 821 individuals (6 339 782 [52.5%] boys). There were 2 309 841 individuals (19.1%) aged 0 to 4 years, 2 951 679 individuals (24.5%) aged 5 to 9 years, 3 489 709 individuals (28.9%) aged 10 to 14 years, and 3 317 593 individuals (27.5%) aged 15 to 19 years. Of these, 1 275 829 individuals (10.6%) were exposed to diagnostic low-dose ionizing radiation between 2006 and 2015, and 10 792 992 individuals (89.4%) were not exposed. By December 31, 2015, 21 912 cancers were recorded. Among individuals who had been exposed, 1444 individuals (0.1%) received a cancer diagnosis. The overall cancer incidence was greater among exposed individuals than among nonexposed individuals after adjusting for age and sex (IRR, 1.64 [95% CI, 1.56-1.73]; *P* < .001). Among individuals who had undergone computed tomography scans in particular, the overall cancer incidence was greater among exposed individuals than among nonexposed individuals after adjusting for age and sex (IRR, 1.54 [95% CI, 1.45-1.63]; *P* < .001). The incidence of cancer increased significantly for many types of lymphoid, hematopoietic, and solid cancers after exposure to diagnostic low-dose ionizing radiation. Among lymphoid and hematopoietic cancers, incidence of cancer increased the most for other myeloid leukemias (IRR, 2.14 [95% CI, 1.86-2.46]) and myelodysplasia (IRR, 2.48 [95% CI, 1.77-3.47]). Among solid cancers, incidence of cancer increased the most for breast (IRR, 2.32 [95% CI, 1.35-3.99]) and thyroid (IRR, 2.19 [95% CI, 1.97-2.20]) cancers.

**Conclusions and Relevance:**

This study found an association of increased incidence of cancer with exposure to diagnostic low-dose ionizing radiation in a large cohort. Given this risk, diagnostic low-dose ionizing radiation should be limited to situations in which there is a definite clinical indication.

## Key Points

**Question:**

Is exposure to diagnostic low-dose ionizing radiation in youths associated with increased risk of cancer?

**Findings:**

In this population-based cohort study including more than 12 million South Korean youths, the overall cancer incidence was greater among individuals exposed to diagnostic low-dose ionizing radiation than among nonexposed individuals after adjusting for age and sex (incidence rate ratio, 1.64). The incidence of cancer increased significantly for many types of cancers after radiation exposure, particularly mouth and pharynx, breast, thyroid, lymphoid and hematopoietic, and myelodysplasia cancers.

**Meaning:**

The association of increased cancer risk with exposure to diagnostic low-dose ionizing radiation may be important to inform decisions about diagnostic use of low-dose ionizing radiation in Asian youth populations worldwide.

## Teaser

This cohort study examines the association of low-dose radiation exposure in youths with risk of cancer using South Korean national health insurance data.

## Introduction

Diagnostic low-dose ionizing radiation has great medical benefits; however, its widespread use has also raised concerns about adverse effects of radiation. The largest concern with ionizing radiation is increased cancer risk, particularly after childhood exposures.^1,2,3,4,5,6,7,8,9,10,11,12,13,14,15^ The biological hazards of ionizing radiation have been well documented since the atomic bomb explosions in Hiroshima, Japan, and Nagasaki, Japan, which caused a high incidence of atomic bomb–induced health issues, including various types of cancer.^16,17,18^ A 2012 study of 180 000 young people who underwent computed tomography (CT) scans in the United Kingdom reported increased risk of leukemia and brain cancer, which was correlated with the dose of radiation.^19^ That study reported provisional risk estimates for these 2 cancers. Unfortunately, there are fewer studies that address cancer risk after exposure to diagnostic low- to medium-dose ionizing radiation, to our knowledge. Previously, it was thought to be impractical to directly estimate the risk of cancer after such low doses of radiation.^19,20,21,22,23^ This study used a representative sample of all South Korean National Health Insurance System (KNHIS) claims data to derive direct estimates of cancer risk associated with diagnostic low-dose ionizing radiation exposure in individuals aged 0 to 19 years by comparing their cancer incidence with that of a comparison cohort.

## Methods

### Database

This study used cohort data released by the KNHIS in 2017. The data were derived from 49 570 064 nationally representative individuals who constitute the entire population in the KNHIS, and include all medical claims filed from 2002 to 2015. All exposures to diagnostic low-dose ionizing radiation funded by the KNHIS during 2002 to 2015 were identified for this cohort. The KNHIS has offered a special support system for rare and intractable disease, including cancers, since 2006. Consequently, more than 90% of medical payments for patients with confirmed cancer diagnoses are supported by insurance. We included individuals with confirmed diagnoses who were being supported by the KNHIS special support system. In addition, to exclude cancer survivors and disease recurrence, we excluded individuals who had received cancer diagnoses prior to the study entry date.

This study adhered to the tenets of the Declaration of Helsinki.^24^ The KNHIS National Sample Cohort project was approved by the institutional review board of the KNHIS. This study design was also reviewed and approved by the institutional review board of Korea University Medical Center, Gyeonggi-do, South Korea. Written informed consent was waived because data were deidentified. This study followed the Strengthening the Reporting of Observational Studies in Epidemiology (STROBE) reporting guideline. Data were analyzed from March 2018 to September 2018.

### Study Design

Participants entered the study on January 1, 2006, and remained in it until the exit date. The exit date was either December 31, 2015, the date of death, or the date of cancer diagnosis. Cancer diagnoses were based on *International Statistical Classification of Diseases and Related Health Problems, Tenth Revision (ICD-10)* codes.^25^ Cancers that were diagnosed in cohort members through December 31, 2015, were assessed if there was at least a 2-year interval between diagnostic low-dose ionizing radiation exposure and diagnosis. Diagnostic low-dose ionizing radiation exposures were defined as any that occurred on or after the entry date, when the participant was aged 0 to 19 years, on or before the exit date, and at least 2 years (lag period) before any cancer diagnosis.^21^ The lag period was adopted given the possibility that the scan was part of a diagnostic evaluation for cancer. To minimize the bias according to lag period, we calculated the risks with 3 different lag periods (1, 2, and 5 years). We set the first exposure date as the start point of the lag period, and multiple exposures were calculated if additional exposures occurred during the lag period. To calculate person-years at risk, we assigned each individual to the nonexposed group from entry until transfer (date of the first exposure plus the lag period). For the exposed group, we assigned individuals from transfer until exit (eFigure 1 in the Supplement). We hypothesized a lag period of 0 to 2 years for hematopoietic cancers and 0 to 5 years for solid tumors.^26,27^ Repeated imaging is recommended for solid tumors, particularly in the early period after clinical symptoms appear or after a tumor is incidentally found on imaging performed for another reason.^27^

### Statistical Analysis

The primary analysis assessed the incidence rate ratios (IRRs) for exposed vs nonexposed individuals using the number of person-years as an offset. We used likelihood ratio tests to assess the significance of IRR departures from unity. The IRRs were calculated using the Poisson regression model after adjusting for age and sex. Cox proportional hazard regression models were used to adjust the different duration and potential dropout. The risks of exposure were calculated with IRRs and the excess number of cancers. These risks were divided into the risks of each cancer according to categorized *ICD-10* code, assuming a Poisson distribution with 95% CI. The floating 95% CI for the IRR categorized according to the number of diagnostic scans was calculated using the amount of information in each category. The procedures were programmed in SAS statistical software version 9.3 (SAS Institute) using 2-tailed *P* values, and statistical significance was set at *P* less than .05. The number of diagnostic low-dose ionizing radiation events provided the simplest measure of a person’s radiation exposure.

## Results

### Study Population and Overall Cancer Risk

The cohort included a total of 12 068 821 individuals (6 339 782 [52.5%] boys) (eFigure 2 in the Supplement). At baseline, there were 2 309 841 individuals (19.1%) aged 0 to 4 years, 2 951 679 individuals (24.5%) aged 5 to 9 years, 3 489 709 individuals (28.9%) aged 10 to 14 years, and 3 317 593 individuals (27.5%) aged 15 to 19 years (Table 1). The distributions of age, sex, income, and place of residence were stratified for statistical analysis. Based on a 2-year lag period, 1 275 829 individuals (10.6%) were transferred into the diagnostic low-dose ionizing radiation exposed group before exit from the study. Of those in the exposed group, 178 518 individuals (14.0%) underwent more than 1 scan. Among the full cohort, 21 912 cancers were recorded by December 31, 2015, including 1444 cancers in 1 275 829 individuals (0.1%) exposed to diagnostic low-dose ionizing radiation at least 2 years before any cancer diagnosis (Table 2). The overall cancer incidence was greater for exposed individuals than it was for nonexposed individuals after adjusting for age and sex (IRR, 1.64 [95% CI, 1.56-1.73]; *P* < .001) (Table 3).

**Table 1.** Characteristics of the Study Population

Characteristic	No. (%)		Total, No. (N = 12 068 821)
	Exposed (n = 1 275 829)^a^	Nonexposed (n = 10 792 99)	
Age at baseline, y			
0-4	277 158 (12.0)	2 032 683 (88.0)	2 309 841
5-9	325 815 (11.0)	2 625 864 (89.0)	2 951 679
10-14	461 140 (13.2)	3 028 569 (86.8)	3 489 709
15-19	211 716 (6.4)	3 105 876 (93.6)	3 317 592
Sex			
Male	791 894 (12.5)	5 547 888 (87.5)	6 339 782
Female	483 935 (8.5)	5 245 104 (91.6)	5 729 039
Income			
High 80%	974 785 (10.5)	8 310 631 (89.5)	9 285 416
Low 20%	301 044 (10.8)	2 482 361 (89.2)	2 783 405
Place of residence			
Urban	552 249 (10.2)	4 845 926 (89.8)	5 398 175
Rural	723 507 (10.9)	5 946 521 (89.2)	6 670 028

^a^ Defined as individuals exposed to diagnostic low-dose ionizing radiation on or after the entry date, when the individual was aged 0 to 19 years, on or before the exit date, and at least 2 years (lag period) before any cancer diagnosis. All individuals included in the study were classified as nonexposed at study entry. Individuals exposed to diagnostic low-dose ionizing radiation continued to be classified as nonexposed for the duration of the lag period, after which they were transferred to the exposed group.

**Table 2.** Characteristics of Individuals Exposed to Diagnostic Low-Dose Ionizing Radiation Based on a 2-Year Lag Period

Characteristic	Individuals, No. (%) (n = 1 275 829)
Age at first exposure, y	
0-4	277 158 (21.7)
5-9	325 815 (25.5)
10-14	461 140 (36.1)
15-19	211 716 (16.6)
Total exposures	
1	1 097 311 (86.0)
2	140 210 (11.0)
3	26 160 (2.1)
4	7285 (0.6)
>5	4863 (0.4)
Type of first exposure	
Computed tomography	1 179 021 (92.4)
Brain or head^a^	584 246 (45.8)
Abdominal^b^	285 554 (22.4)
Spine or neck	110 561 (8.7)
Chest	88 189 (6.9)
Extremity	110 471 (8.7)
Intravenous urography	30 445 (2.4)
Upper gastrointestinal series	21 294 (1.7)
Bone scan	20 108 (1.6)
Others (interventions with low-dose radiation)^c^	24 961 (2.0)

^a^ Includes the brain or head in combination with other sites.

^b^ Includes combined scans of the abdomen, chest, or pelvis.

^c^ Includes cardiac resting ventriculography, diagnostic cardiac catheterization, myocardial perfusion imaging, percutaneous coronary intervention, percutaneous brain intervention, diagnostic head and neck angiography, diagnostic chest angiography, diagnostic chest angiography, thyroid uptake, and positron emission tomography.

**Table 3.** Outcomes of Individuals Exposed to Diagnostic Low-Dose Ionizing Radiation Group by Cancer Type Based on a 2-Year Lag Period^a^

Cancer Type	No.				Incidence Rate Ratio (95% CI)
	Observed Cancers			Excess Cancers	
	Exposed Group^b^	Nonexposed Group	Total		
Solid malignant neoplasm	987	14 327	15 314	404.8	1.70 (1.59-1.81)
Mouth and pharynx	30	350	380	15.1	2.01 (1.38-2.92)
Digestive	76	898	974	39.0	1.83 (1.22-2.74)
Respiratory	35	396	431	17.0	1.95 (1.38-2.75)
Bone	53	1070	1123	2.3	1.05 (0.79-1.38)
Melanoma	14	248	262	3.4	1.32 (0.77-2.26)
Soft tissue	43	778	821	7.1	1.20 (0.88-1.63)
Breast	14	225	239	8.0	2.32 (1.35-3.99)
Female genital	76	1309	1385	33.2	1.77 (1.41-2.24)
Male genital	23	354	377	5.0	1.28 (0.84-1.95)
Urinary	20	366	386	2.7	1.16 (0.74-1.82)
Brain	183	2689	2872	53.0	1.57 (1.38-1.78)
Thyroid	363	4862	5225	197.4	2.19 (1.97-2.44)
Unspecified	57	782	839	23.2	1.68 (1.29-2.20)
Lymphoid and hematopoietic malignant neoplasm	457	6141	6598	159.1	1.53 (1.39-1.69)
Hodgkin	21	364	385	5.1	1.32 (0.85-2.05)
Other lymphomas	47	552	599	19.8	1.73 (1.28-2.32)
Other lymphoid	57	967	1024	12.2	1.27 (0.97-1.66)
Leukemia and myeloid	332	4258	4590	122.4	1.58 (1.42-1.77)
Leukemia	294	3924	4218	99.9	1.51 (1.34-1.71)
Lymphoid leukemia	74	1805	1879	−17.3	0.81 (0.64-1.02)
Other myeloid	220	2119	2339	117.1	2.14 (1.86-2.46)
Myelodysplasia	38	334	372	22.7	2.48 (1.77-3.47)
Total	1444	20 468	21 912	565.0	1.64 (1.56-1.73)

^a^ Cancer type classified by *International Statistical Classification of Diseases and Related Health Problems, Tenth Revision*. Incidence rate ratios and number of excess cancers were calculated compared with the nonexposed group after stratification by age and sex.

^b^ Defined as individuals exposed to diagnostic low-dose ionizing radiation on or after the entry date, when the individual was aged 0 to 19 years, on or before the exit date, and at least 2 years (lag period) before any cancer diagnosis. All individuals included in the study were classified as nonexposed at study entry. Individuals exposed to diagnostic low-dose ionizing radiation continued to be classified as nonexposed for the duration of the lag period, after which they were transferred to the exposed group.

### Cancer Risk Associated With CT Scan

Based on the 2-year lag period, a total of 1 179 021 individuals (9.8%) were transferred into the CT-exposed group before exit from the study. Among the full cohort, 21 912 cancers were recorded by December 31, 2015, including 1216 cancers in 1 179 021 individuals (0.1%) exposed to CT at least 2 years prior to any cancer diagnosis. The overall cancer incidence was greater for exposed individuals than it was for nonexposed individuals after adjusting for age and sex (IRR, 1.54 [95% CI, 1.45-1.63]; *P* < .001) (Table 4).

**Table 4.** Outcomes of Individuals Exposed to Computed Tomography by Cancer Type Based on a 2-Year Lag Period^a^

Cancer Type	No.				Incidence Rate Ratio (95% CI)
	Observed Cancers			Excess Cancers	
	Exposed Group^b^	Nonexposed Group	Total		
Solid malignant neoplasm	840	14 474	15 314	321.0	1.62 (1.51-1.74)
Mouth and pharynx	29	351	380	15.7	2.19 (1.50-3.20)
Digestive	65	909	974	32.0	1.97 (1.53-2.53)
Respiratory	34	397	431	18.0	2.01 (1.46-2.78)
Bone	47	1076	1123	1.3	1.03 (0.77-1.38)
Melanoma	13	249	262	3.6	1.38 (0.79-2.41)
Soft tissue	40	781	821	7.8	1.24 (0.90-1.71)
Breast	13	226	239	7.9	2.53 (1.44-4.43)
Female genital	71	1314	1385	33.9	1.92 (1.51-2.43)
Male genital	22	355	377	5.8	1.36 (0.88-2.09)
Urinary	18	368	386	2.4	1.15 (0.72-1.85)
Brain	166	2706	2872	48.3	1.55 (1.36-1.77)
Thyroid	273	4952	5225	126.9	1.87 (1.65-2.11)
Unspecified	49	790	839	18.6	1.61 (1.21-2.15)
Lymphoid and hematopoietic malignant neoplasm	376	6222	6598	104.2	1.38 (1.25-1.54)
Hodgkin	20	365	385	5.9	1.42 (0.90-2.23)
Other lymphomas	41	558	599	16.2	1.66 (1.20-2.27)
Other lymphoid	49	975	1024	8.7	1.22 (0.91-1.62)
Leukemia and myeloid	266	4324	4590	73.9	1.38 (1.22-1.57)
Leukemia	233	3985	4218	54.9	1.31 (1.15-1.49)
Lymphoid leukemia	68	1811	1879	−15.0	0.82 (0.64-1.04)
Other myeloid	165	2174	2339	69.9	1.73 (1.48-2.03)
Myelodysplasia	33	339	372	19.1	2.38 (1.66-3.40)
Total	1216	20 696	21 912	426.3	1.54 (1.45-1.63)

^a^ Cancer type classified by *International Statistical Classification of Diseases and Related Health Problems, Tenth Revision*. Incidence rate ratios and number of excess cancers were calculated compared with the nonexposed group and calculated after stratification by age and sex.

^b^ Defined as individuals exposed to diagnostic low-dose ionizing radiation on or after the entry date, when the individual was aged 0 to 19 years, on or before the exit date, and at least 2 years (lag period) before any cancer diagnosis. All individuals included in the study were classified as nonexposed at study entry. Individuals exposed to diagnostic low-dose ionizing radiation continued to be classified as nonexposed for the duration of the lag period, after which they were transferred to the exposed group.

### IRRs for Various Lag Periods

Incidence rate ratios decreased with longer lag period (Table 5), but the difference was not statistically significant. Consequently, we used IRRs with a 2-year lag period for further analysis.

**Table 5.** Number of Cancers and IRRs for Various Lag Periods Among Individuals Exposed to Diagnostic Low-Dose Ionizing Radiation^a^

Lag	No.				IRR (95% CI)	Excess Cancers, No.
	Exposed Group^b^		Nonexposed Group			
	Cancers	Person-Years	Cancers	Person-Years		
1 y	1921	5 848 313	19 991	95 751 152	1.72 (1.64-1.80)	804.4
2 y	1444	4 499 100	20 468	97 109 854	1.64 (1.56-1.73)	565.0
5 y	434	1 442 916	21 478	101 918 397	1.48 (1.35-1.63)	141.0

Abbreviation: IRR, incidence rate ratio.

^a^ The IRR and number of excess cancers are compared with the nonexposed group and calculated after stratification by age and sex.

^b^ Defined as individuals exposed to diagnostic low-dose ionizing radiation on or after the entry date, when the individual was aged 0 to 19 years, on or before the exit date, and at least 2 years (lag period) before any cancer diagnosis. All individuals included in the study were classified as nonexposed at study entry. Individuals exposed to diagnostic low-dose ionizing radiation continued to be classified as nonexposed for the duration of the lag period, after which they were transferred to the exposed group.

### Risks of Specific Cancers

There was an excess of 404.8 solid cancers and 159.1 lymphoid and hematopoietic cancers in individuals exposed to diagnostic low-dose ionizing radiation compared with in those who were nonexposed (Table 3). The incidence of cancer increased significantly for many types of lymphoid and hematopoietic cancers (IRR, 1.53 [95% CI, 1.39-1.69]), as well as for solid cancers (IRR, 1.70 [95% CI, 1.59-1.81]). Among lymphoid and hematopoietic cancers, myelodysplasia (IRR, 2.48 [95% CI, 1.77-3.47]) and other myeloid leukemias (IRR, 2.14 [95% CI, 1.86-2.46]) had the highest incidence, although the incidence was also increased in other cancers. Among solid cancers, breast (IRR, 2.32 [95% CI, 1.35-3.99]), thyroid (IRR, 2.19 [95% CI, 1.97-2.20]), and mouth and pharynx (IRR, 2.01 [95% CI, 1.38-2.92]) had the highest incidence, although the incidence was also increased in other solid cancers. We found similar results in the CT exposure group (Table 4).

### Cancer Risk According to Type of First Exposure

Lymphoid and hematopoietic cancers consistently had higher IRRs with various amounts of irradiation. The IRR of the exposed group was higher than that of the nonexposed group not only in the target area of irradiation, eg, the IRR for brain cancer was higher among individuals who underwent head or brain CT than among the overall group exposed to any diagnostic low-dose ionizing radiation (1.65 [95% CI, 1.35-2.01] vs 1.57 [95% CI, 1.38-1.78]) but also for lymphoid and hematopoietic cancers when considered separately among individuals who underwent bone scans (3.25 [95% CI, 1.46-7.23]) (eTable 1 in the Supplement). In addition, compared with the total group exposed to diagnostic low-dose ionizing radiation, there was a higher IRR among individuals exposed to abdominal imaging for digestive (3.11 [95% CI, 2.10-4.59] vs 1.83 [95% CI, 1.22-2.74]), breast (3.35 [95% CI, 1.49-7.54] vs 2.32 [95% CI, 1.35-3.99]), and female genital (2.62 [95% CI, 1.85-3.72] vs 1.77 [95% CI, 1.41-2.24]) cancers. Chest CT was associated with significantly increased IRR for respiratory cancer compared with the full diagnostic low-dose ionizing radiation exposure group (5.68 [95% CI, 2.93-11.01] vs 1.95 [95% CI, 1.38-2.75]). Mouth and pharynx cancer had higher IRR with spine or neck CT than with other CT exposures (6.46 [95% CI, 3.45-12.11] vs 2.19 [95% CI, 1.50-3.20]) (eTable 1 in the Supplement).

### Cancer Risk According to Number of Exposures

The incidence of cancer increased significantly with additional diagnostic low-dose radiation exposures (eTable 2 in the Supplement). We compared the different IRRs of 3 lag periods to minimize the reverse causation of certain cancers. Individuals with more than 3 CT scans had significantly increased IRRs in 3 various lag periods (1 year: 9.05 [95% CI 7.84-10.46]; 2 years: 5.98 [95% CI, 5.13-6.98]; 5 years: 2.90 [95% CI, 2.19-3.83]), although the number of individuals was insufficient to determine the associations of repeated scans.

## Discussion

### Overall Associations of Diagnostic Low-Dose Ionizing Radiation Exposure Among Young People

The increased use of CT in general has resulted in many children receiving high-dose examinations.^28^ If the carcinogenic effect of diagnostic low-dose radiation is greater in a subset of people who are genetically susceptible, it would have important clinical implications for the standards of radiation protection.^2,5,10,13,14,15,29,30,31^ Various studies have used risk projection models to estimate the potential lifetime excess cancer risk from CT scans. These models are largely based on risk models from studies of survivors of the atomic bombs in Japan.^16,17,18^ Our study found an association of increased cancer incidence with exposure to ionizing radiation and with exposure to CT scans. Therefore, our findings raise concerns regarding the use and subsequent risks of diagnostic low-dose ionizing radiation exposure in youths.^3,8,9,14,31,32^ After adjusting for age and sex, the overall cancer incidence was greater in individuals exposed to radiation than it was in those who were nonexposed. However, risk analysis in the United States suggests that, for children, the lifetime excess risk of any incident cancer for a head CT scan is approximately 1 cancer per 1000 to 2000 scans.^1,19^ Therefore, the absolute excess lifetime cancer risk is small compared with the lifetime risk of developing cancer in the general population, which is approximately 1 in 3.^19^ Provided that imaging is clinically justified, it might be appropriate in a younger patient who needs correct diagnosis.

### Risks of Specific Cancers

In a 2012 UK cohort study,^19^ significant associations were found between the estimated radiation doses to the red bone marrow and brain provided by CT scans and the incidence of leukemia and brain tumors. The study by Pearce et al^19^ reported that the cumulative ionizing radiation doses from 2 to 3 head CTs could increase the risk of brain tumors nearly 3-fold, while 5 to 10 head CTs could increase the risk of leukemia 3-fold. In a 2013 Australian cohort study,^21^ there were increased risks of several types of solid cancers and of leukemia, myelodysplasia, and other lymphoid cancers among individuals exposed to at least 1 CT scan compared with nonexposed young people. Our results are similar to those of these studies.^19,21^ There were 404.8 additional solid cancers and 159.1 additional lymphoid or hematopoietic cancers in individuals who had been exposed to diagnostic low-dose ionizing radiation compared with those nonexposed. This association was also true for solid cancers and lymphoid and hematopoietic cancers when considered separately. Among lymphoid and hematopoietic cancers and other lymphomas, myeloid leukemias and myelodysplasia had the largest IRRs. However, the incidence was also increased in other cancers. Among solid cancers, the largest IRRs were present in mouth and pharynx, breast, and thyroid cancers. These results were similar to the conclusions of the 2008 United Nations Scientific Committee on the Effects of Atomic Radiation study,^26^ which reported a positive association between mouth and pharynx, respiratory, breast, and thyroid cancers, as well as leukemias, after low-dose ionizing radiation exposure. A 2016 study^33^ reported that children exposed to low-dose diagnostic ionizing radiation had an increased likelihood of developing leukemia compared with those who were not exposed.

### Cancer Risk According to Radiation Type

A study by Miglioretti et al^28^ evaluated trends of CT use in pediatrics, as well as the association of radiation exposure with cancer risk. They reported that attributable risk was higher in patients who underwent CT scans of the abdomen and pelvis or spine than in those who underwent other types of CT. In our study, lymphoid and hematopoietic cancers had consistently higher IRR values compared with other cancers. In addition, digestive, female genital, and breast cancers demonstrated higher IRR values with abdominal CT than with other types of CT. Similarly, mouth and pharynx cancers had a higher IRR associated with spine or neck CT than with other diagnostic radiation types. These trends are similar to those of previous studies,^21,28^ which emphasize dose reduction strategies with regard to specific types of radiation and populations. However, one should be cautious when interpreting these results given the lack of clinical information regarding the reasons for using diagnostic radiation. Although we observed a similarly increased pattern in the 5-year lag period with specific types of radiation, we cannot completely rule out reverse causation. In addition, a study by Nikkila et al^33^ reported that background low doses of ionizing radiation were associated with increased risk of childhood leukemia. In the study by Nikkila et al,^33^ collected survey data of background gamma radiation in Finland were used to assess the background exposure of these study individuals. If we consider other variables (reverse causation and background low-dose radiation), we cannot determine the true associations of certain kinds of diagnostic radiation.

### Comparison With Other Studies

We recorded 21 912 cancers, including 1444 cancers in 1 275 829 individuals exposed to diagnostic low-dose radiation at least 2 years prior to any cancer diagnosis. The overall cancer incidence was greater in individuals who had been exposed to a CT scan than in nonexposed individuals after adjusting for age and sex. In a 2013 Australian study,^21^ 60 674 cancers were recorded, including 3150 in 680 211 individuals who had been exposed to a CT scan at least 1 year before cancer diagnosis. The overall cancer incidence was greater in individuals who had been exposed than in nonexposed individuals after accounting for age, sex, and birth year. These results are similar to a 2012 study^19^ of young people exposed to CT scans in the United Kingdom. In our study, the overall cancer incidence was smaller than that in other studies. Although ethnic and regional differences should also be considered, this result suggests that the cancer diagnoses in this study were accurate, which could minimize overestimation or underestimation of the carcinogenic effect of radiation. Furthermore, our study found that exposure to other forms of diagnostic low-dose ionizing radiation, including intravenous urography, upper gastrointestinal tract series, bone scan, and others, were also associated with increased IRRs.^4,19,21,32^ It is important not to overestimate the effect of CT, and all forms of diagnostic low-dose ionizing radiation should be assessed. In addition, we calculated risks of 3 different lag periods to minimize reverse causation according to previous investigations.^26,27^ Although we adopted a 2-year lag period after comparison of the IRRs for different lag periods, 1-year and 5-year lag periods were associated with similar IRR increases, which supports the reliability of our study.

### Strengths and Limitations

Our study had several strengths. To our knowledge, it was one of the largest population-based studies to date that evaluated diagnostic medical radiation exposure. The data were obtained from 49 570 064 nationally representative individuals.^34,35,36^ In South Korea, patients with KNHIS pay 30% of their total medical expenses, while the medical providers are required to submit claims for the remaining 70%. These claims are accompanied by the direct medical costs of both inpatient and outpatient care. A total of 97% of the South Korean population is covered by the Medical Assistance Program. Therefore, nearly all of the data in the health system are centralized in large databases. None of the patients’ health care records were duplicated or omitted, because all South Korean residents receive a unique identification number at birth. Public funding under the comprehensive health insurance system has allowed us to study the exposed cohort drawn from the general population.^34,35,36^ We believe that this sample is likely representative of the population of children and young adults in South Korea who have undergone diagnostic low-dose ionizing radiation. To our knowledge, this is the first study to explore the cancer risk associated with overall diagnostic low-dose ionizing radiation in an Asian cohort. Our results contribute to the literature for decision-making regarding the diagnostic use of low-dose ionizing radiation in young populations in Asia and worldwide.

This study also has several limitations. It was not possible to collect protocols or machine parameters for all of the exposures; therefore, we were unable to estimate individual radiation doses. Consequently, we could not calculate a direct estimate of the excess rate ratio per gray, as did other studies from the United Kingdom^19^ and Australia.^21^ Our study would have missed exposures that took place outside of South Korea, before January 1, 2002, after December 31, 2015, and those in patients older than 19 years. We adopted floating CIs, which are sensitive and result in overly narrow interval estimates.^37^ A major weakness of our study was a lack of information regarding the reasons for obtaining a CT. We cannot assume that all of the excess cancers observed during the follow-up period were associated with low-dose ionizing radiation, as scanning decisions were based on medical indications.^38^ Some of these indications may confound the association if they were also associated with cancer. If these factors are available in the claims data, then they should be included for statistical adjustment. Therefore, we cannot rule out the possibility of reverse causation, in which the early symptoms of cancer prompted exposure to diagnostic low-dose radiation. A study by Walsh et al^38^ reported that the published reports of CT scan studies suggest that their findings should be interpreted with caution given the potential for reverse causation. They reported that it is difficult to extract conclusions regarding the risk of radiation exposure given the considerable number of extraneous factors related to the reasons for performing CT scan. We agree with the criticisms of our study design and recognize the possibility of reverse causation in our results. Another source of confounding may include geographic factors. Recently, several study designs have been adopted to overcome similar biases of previous study designs. A study by Berrington de González et al^39^ reanalyzed the original data and reviewed additional clinical information from radiology information systems databases, including the underlying cause of death and pathology reports. Interestingly, the study by Berrington de González et al found similar results after these additional analyses.^39^ Additionally, we could not completely adjust the possible extra-Poisson variation, but negative binomial models or Poisson with robust SEs can be used to handle it. Statistical adjustment with a different method may improve the accuracy of IRRs in a future study.

## Conclusions

In conclusion, the associations we found of diagnostic low-dose ionizing radiation with increased incidence of cancer in youths suggest that there is incentive to limit radiation doses to as low as reasonably achievable and to only scan when justified.^29,40^ Medical professionals should weigh the benefits of diagnostic low-dose ionizing radiation with the associated risks to justify each decision.

## Notes

Supplement. **eFigure 1.** Summary of the Study Population **eFigure 2.** Schematic Diagram of How Participants Were Included in Exposed and Nonexposed Groups **eTable 1.** Outcomes of the Exposed Group Stratified by Cancer Type According to Type of First Exposure **eTable 2.** Outcomes of the Exposed Group Stratified by Cancer Type According to Number of Exposures to Computed Tomography
