# Supplementary material for: Association of Exposure to Diagnostic Low-Dose Ionizing Radiation With Risk of Cancer Among Youths in South Korea
Source: JAMA Netw Open. 2019 Sep 4;2(9):e1910584. doi: 10.1001/jamanetworkopen.2019.10584 (PMC6727680; doi:10.1001/jamanetworkopen.2019.10584)
Supplement: Supplement. — eFigure 1. Summary of the Study Population eFigure 2. Schematic Diagram of How Participants Were Included in Exposed and Nonexposed Groups eTable 1. Outcomes of the Exposed Group Stratified by Cancer Type According to Type of First Exposure eTable 2. Outcomes of the Exposed Group Stratified by Cancer Type According to Number of Exposures to Computed Tomography [file jamanetwopen-2-e1910584-s001.pdf]

Supplementary Online Content

Hong J-Y, Han K, Jung J-H, Kim JS. Association of exposure to diagnostic low-dose ionizing radiation with risk of cancer among youths in South Korea. *JAMA Netw Open*. 2019;2(9):e1910584. doi:10.1001/jamanetworkopen.2019.10584

eFigure 1. Summary of the Study Population

eFigure 2. Schematic Diagram of How Participants Were Included in Exposed and Nonexposed Groups

eTable 1. Outcomes of the Exposed Group Stratified by Cancer Type According to Type of First Exposure

eTable 2. Outcomes of the Exposed Group Stratified by Cancer Type According to Number of Exposures to Computed Tomography

This supplementary material has been provided by the authors to give readers additional information about their work.

© 2019 Hong J-Y et al. *JAMA Network Open.*

eFigure 1. Summary of the Study Population

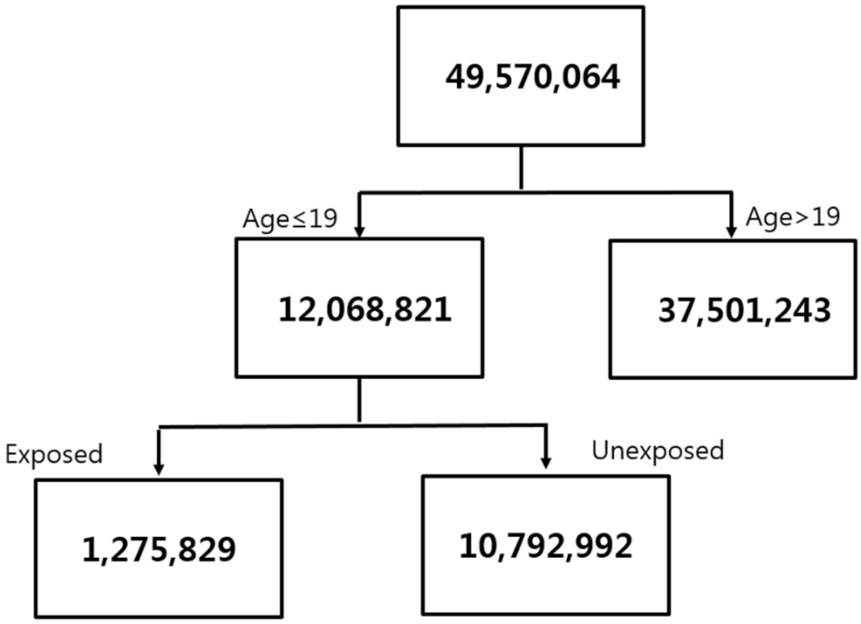

© 2019 Hong J-Y et al. *JAMA Network Open.*

eFigure 2. Schematic Diagram of How Participants Were Included in Exposed and Nonexposed Groups

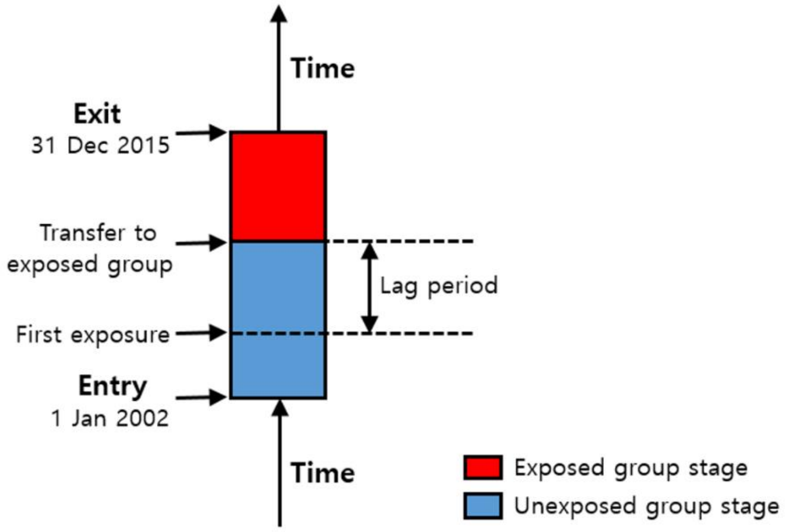

© 2019 Hong J-Y et al. *JAMA Network Open.*

eTable 1. Outcomes of the Exposed Group Stratified by Cancer Type According to Type of First Exposure

Type of exposure	Cancer types (ICD-10)	No of cancers		Excess Ca	IRR (95% CI)
		Exposed	Unexposed		
**Head & Brain CT**	**Total**	539	21,373	167.9	1.45 (1.33 to 1.58)
	**Brain**	102	2,770	40.2	1.65 (1.35 to 2.01)
**Abdominal CT**	**Total**	328	21,584	137.2	1.72 (1.54 to 1.92)
	**Digestive**	26	948	17.6	3.11 (2.10 to 4.59)
	**Breast**	6	233	4.2	3.35 (1.49 to 7.54)
	**Fe genital**	32	1,353	19.8	2.62 (1.85 to 3.72)
**Chest CT**	**Total**	110	21,802	44.3	1.67 (1.39 to 2.02)
	**Respiratory**	9	422	7.4	5.68 (2.93 to 11.01)
**Spine or neck CT**	**Total**	156	21,756	70.8	1.83 (1.56 to 2.14)
	**Mouth & pharynx**	10	370	8.5	6.46 (3.45 to 12.11)
	**Thyroid**	44	5,181	23.2	2.12 (1.57 to 2.85)
**Extremity CT**	**Total**	83	21,829	26.2	1.46 (1.18 to 1.81)
	**Soft**	6	815	3.7	2.57 (1.15 to 5.73)
**IV urography**	**Total**	21	21,885	-3.5	0.88 (0.61 to 1.29)
	**Urinary**	0	386	-	-
**Upper GI series**	**Total**	30	21,882	9.3	1.45 (1.01 to 2.08)
	**Digestive**	3	971	2.1	3.22 (1.04 to 10.01)
**Bone scan**	**Total**	15	21,897	8.9	2.46 (1.48 to 4.08)
	**L & hemato†**	6	6,592	4.2	3.25 (1.46 to 7.23)

IRR=incidence rate ratio, exposed vs. unexposed

IRR and number of excess cancers calculated after stratification by age and sex.

†L & hemato=lymphoid & hematopoietic

© 2019 Hong J-Y et al. *JAMA Network Open.*

eTable 2. Outcomes of the Exposed Group Stratified by Cancer Type According to Number of Exposures to Computed Tomography

	Number of exposures					
	1		2		≥3	
**Lag**	**No of cancers**	**IRR (95% CI)**	**No of cancers**	**IRR (95% CI)**	**No of cancers**	**IRR (95% CI)**
**1 year**	1,496	1.49 (1.41 to 1.57)	238	2.62 (2.31 to 2.98)	187	9.05 (7.84 to 10.46)
**2 years**	1,088	1.44 (1.45 to 1.53)	194	2.03 (1.77 to 2.34)	162	5.98 (5.13 to 6.98)
**5 years**	323	1.39 (1.24 to 1.55)	62	1.44 (1.12 to 1.84)	49	2.90 (2.19 to 3.83)

IRR=incidence rate ratio, exposed vs. unexposed
IRR calculated after stratification by age and sex.

© 2019 Hong J-Y et al. *JAMA Network Open.*
